# Medicine

**Published:** 1906-09

**Authors:** J. Michell Clarke


### Progress of the Medical Sciences.

### MEDICINE.

The well-known **cumulative action of digitalis** is to be explained, according to Focke,[1] as a total effect due to the combination of the action of several single doses. Such a combined effect is rendered possible by the slow absorption and

[1] *Med. Klinik*, 31, 1905.

slow elimination of the drug; a further factor lies in the great variation in the strength of similarly-named preparations. The absorption of a single full dose is completed in the following periods: For pure digitoxin, in four days; for the powdered digitalis leaves, in about two; and for the tincture, in one day. If these intervals have elapsed after the last dose, no stronger effect than that which may be already evident is to be expected; if the effect produced is not sufficient after these intervals for each preparation respectively, the drug can then be given anew. The total dose should be a full one, given within a short period of time; if this does not produce the required effect, the second total dose should be smaller than the first. As to the duration of excretion, in the case of a full dose of digitoxin given in divided doses spread over several days excretion is completed by eight to ten days afterwards; in the case of an active powder of digitalis leaves, the time is ordinarily fourteen to twenty days. After these periods the amount of the digitalis bodies remaining in the circulation is so small that a maximum dose can again be administered without fear of a cumulative effect.

The want of uniformity in the strength of digitalis preparations is a cause of toxic accumulation, but is avoided by only using preparations guaranteed by the makers to be of constant strength. The leaves act more quickly and the effects last longer than digitoxin.

**Digalen** (digitoxinum solubile. Cloetta) has, according to Haberfeld,[1] the same qualitative effects as digitalis leaves. It does not upset the stomach. begins to act more quickly than the leaves, and has no cumulative result. It produces marked diuresis, and will therefore be quickly eliminated. It is of constant composition. and accordingly can be accurately dosed. Other observers confirm the above statements as to digalen, Pesci saying that it is the best preparation of digitalis for intravenous injection.

**In adherent pericardium,** in which there are clinical signs of adhesion of the heart to the anterior chest-wall, the heart's work is greatly increased by the drawing inwards at each systole of a large part of the bony chest-wall, and the result is that after a longer or shorter time death follows from heart failure. In the operation of cardiolysis the chest-wall overlying the heart is separated from the pericardium and a portion of it removed, so that a soft covering is substituted for the unyielding bony wall which the heart had to drag in at each systole, and its work is thus relieved. Umber[2] has published a case of chronic adhesive mediastino-pericarditis, in which, after cardiolysis, the disturbances of compensation present before the operation disappeared, and the patient recovered his health. He points out that to obtain a good result it is especially important that an endeavour should be made to ascertain before

[1] *Fortschr. d. Med.*, 1905, xxiii. 28. [2] *Therap. d. Gegenw.*, 1905, xlvi. 1.

operation whether the heart muscle is diseased, and if so, to what extent.

**For tapping the pericardium** in serous, serofibrinous, or hemorrhagic effusions Curschmann[1] uses a flat, sheath-shaped trocar, with a stilet ending in a double-edged lancet point. The canula is very thin and makes a very small wound. Aspiration of the fluid is by an attached syphon-tube. The seat of puncture varies according to the conditions present in the individual case. Curschmann places it much farther to the left and outwards than is the custom. In moderate effusions the puncture is made in the left mammary line, in large ones more or less farther outside it. The position is determined at a spot outside the outermost limit of the pericardial friction, which is probably still to be heard outside the apex-beat and within the outer limit of the cardiac dulness. If there is no essential change in the level of the diaphragm, the fifth is the intercostal space chosen ; if the diaphragm is lower than normal, the sixth. So much of the fluid is removed as will flow out by syphonage.

Von Basch, in some critical considerations on the **cardiac rhythm and on arrhythmia,**[2] expresses himself against the compensatory hypothesis initiated by Traube. According to his own view, the important factor in the relative state of well-being, which is established as a secondary condition in patients with heart disease, is not referable to the pathological sequelæ of heart failure, such as hypertrophy and dilatation of parts of the heart. The conditions of well-being in such patients depend much more on the state of the cardiac muscular and nervous apparatus, so that the magnitude of the cardiac contractions can be adapted to its content on the one hand and to the vascular resistance on the other, and thus the stretching of the cardiac walls is not out of proportion to the larger blood-content of the heart cavities brought about during diastolic dilatation. From the form of the valves and the nature of the valvular mechanism the amount of the regurgitated blood in valvular insufficiency can seldom reach a high grade. Von Basch endeavours to show that the conception of the compensatory nature of the long pause, which follows an extra systole, as well as the idea of the ability of the heart to maintain its own rhythm, have a purely hypothetical value. He relies neither upon the "myogenic" theory, nor is he an unconditional supporter of the "neurogenic" theory of the heart's contractions. After Engelmann and Gaskell, not only has the heart's apex, which is free from ganglion cells, the property of rhythmic action, but the rhythmic contraction of the whole heart is to be referred to a peculiar property of the heart muscle itself. On the other hand, von Basch thinks it reasonable to suppose that the fine network of nerves which surrounds the muscle of the apex may have the function of a reflex apparatus, and that

[1] *Therap. d. Gegenw.*, 1905, xlvi. 8, 9. [2] *Arch. f. Physiol.*, 1905, ci, 11, 12.

the contraction of the resting heart's apex may be brought about through indirect irritation. The nature of the physiological stimulus which causes the heart's rhythmic activity we are not in a position to define, and to talk of this normal rhythmic contractility as automatic is only another way of saying that the conditions underlying it are unknown. He thinks it probable, relying on some former investigations, that the physiological stimuli for the cardiac rhythms are not powerful single ones, but weak and discontinuous, and act by summation.

**Forms of arrhythmia.**—Salaghi discusses the nature of various disturbances of the cardiac rhythm.[1] The view generally held as to the cause of the appearance of extra systoles is to attribute them to a want of relation between the power of the heart and the work it has to perform. From the character of the heart affection present it is possible to estimate in some degree in what portion of the heart the pathological extra stimulus is acting, *i.e.* whence the extra systole proceeds. The stimulus which causes a premature contraction may affect the ventricles, the auricles, or the great veins opening into them. According to the seat of disturbance there will be some differences in the time relations of the two contractions (premature and following contraction). If the two contractions together occupy an interval of time corresponding exactly to two beats of the heart the pathological cause is not to be sought in the great veins, but the absence of any compensatory pause points to the latter, and a shortened time interval (for the two beats) to the part of the heart lying above the ventricles as the starting-point of the irritation. If the premature beat takes origin from the right ventricle, auricles or venous ostia, the left ventricle, which will have completely emptied itself during the previous beat, will contract before it has been completely filled. The pulse-wave corresponding to the extra systole will accordingly be proportionately small. This extra pulse-wave will vary as a function of the ventricular diastole, and the small size of the extra pulse is a constant character of this type of extra systole. The corresponding heart's action is not thereby proportionately lessened, so that the appearance of a pseudo-hemi-systole may be given. With the lengthening of the short diastole the pulse may approach more or less to the form of the pulsus alternans.

In direct opposition to this is another type of extra systole. If the resistance to the emptying of the left ventricle is increased, the volume of the blood-wave, driven into the arteries by the force of the ventricular contraction, will depend upon the relation of this force to the concomitant aortic pressure. The size of the extra pulse-wave will then vary with the ventricular systole. Great variability in the pulse-wave of the extra systole

[1] *Berl. Klinik*, 1904, Hft. 192, 1.

is therefore to be expected. From a wave reaching or exceeding the highest pulse-wave there are all degrees to a complete intermission of the pulse.

**Heart affections of scarlet fever.**—Schmaltz, in 191 cases of scarlet fever, found in 35 per cent. some disturbance of the circulatory system.[1] The rapid pulse of the onset has no bearing on the condition of the heart, but the rate of the pulse in the next following stage is of greater significance. As a rule the pulse-rate returns to normal with the fall of temperature, or for a short time is below the rate proportionate to the fever. In a certain number of the latter cases this fall of the pulse is a striking feature, for it may show a marked drop whilst the temperature remains high, or may become too slow. The fall in the pulse-rate sometimes takes place suddenly, at others by gradual stages. If this fall is pronounced, it is preceded or accompanied by other changes in the circulatory apparatus. The bradycardia which follows the slowing of the pulse in the third period always indicates, if it is strongly pronounced, disease of the heart. It is not very unusual for a soft systolic murmur to appear in the first stage of fever, but as a rule murmurs are first heard at a later period. Often a murmur is merely transitory, or it comes and goes, being absent for some days or even for some hours. The systolic murmur is generally accompanied by an accentuation of the pulmonary second sound. Schmaltz noticed the heart to enlarge in 14 per cent. of cases. Arrhythmia frequently accompanies the heart disturbance. Subjective phenomena are as a rule inconspicuous. It is noteworthy that heart troubles do not necessarily accompany severe attacks of scarlet fever, but are more often found after mild attacks. These heart affections have no connection with the so-called scarlatinal rheumatism. Their course is very variable; in a large proportion of cases they soon disappear; in another series the abnormal heart phenomena remain until the recovery of the patient (generally between the forty-first and fifty-fifth days, but often much later). In sixteen out of twenty-four cases who showed cardiac changes during the illness and were examined at much later periods there was pronounced mitral insufficiency. In comparison with diphtheria, the cardiac disturbances of scarlet fever appear to lead more frequently to permanent heart disease. Schmaltz, in a further paper on the condition of the circulatory system in infective diseases generally, points out that although pathological changes are well known during the acute stage, the frequency of their appearance during convalescence is not sufficiently recognised. As a rule, pulse-rate and temperature fall together, the former sometimes remarkably suddenly. Whilst in most cases the pulse-rate then remains normal, in

[1] *München. med. Wchnschr.*, 1904, li. 32.
[2] *Deutsches Arch. f. klin. Med.*, 1905, lxxxv. 10.

others it becomes too slow or again increases in rapidity. This later rapidity is accompanied by softness of the pulse. Irregularity of pulse is the most frequent, and often the first sign of heart disturbance. Examination of the heart may detect nothing abnormal, or accentuation of the pulmonary second sound, galloping, or embryonic rhythm may be heard. Murmurs often appear during the stage of fever, but still more often later, and are frequently transitory. The underlying pathological cause of these disturbances is affection of the heart muscle. As regards prognosis, cases which clinically appear severe may make a complete recovery; in others certain changes may persist for years, or under the influence of the stress of daily life present new and undoubted signs of heart disease.

**Aortic insufficiency has been experimentally produced** in dogs by Ferranini[1] in order to study the relations between the murmur and the second sound in aortic disease, with the result that he found a close correspondence between the auscultatory phenomena and the anatomical extent of the lesion. In the slightest degrees of aortic insufficiency there is simply a want of clearness (sharpness) in the aortic second sound, whilst in the most severe forms a diastolic murmur entirely replaces the sound. Cases of moderate severity form an intermediate group in which both a murmur and a second sound are present, the sound being muffled proportionally to the anatomical extent of lesion. A murmur which runs through the diastole indicates an extensive valvular lesion. If the lesion is not severe, the murmur always *precedes* the remnant of the second sound.

J. MICHELL CLARKE.

NOTE.—For above extracts I am indebted to Schmidt's *Jahrbücher*, 1906, Hft. 5 and 6; Forstmann, *Bericht über neuere Arbeiten auf dem Gebiete der Physiologie und Pathologie des Herzens.*

[1] *Zeitschr. f. Heilk.*, 1903, xxiv. 8.

